# COVID‐19 and its enduring influence on medical imaging

**DOI:** 10.1002/jmrs.608

**Published:** 2022-07-20

**Authors:** Peter O'Reilly, Warren Reed, Sarah Lewis

**Affiliations:** ^1^ Discipline of Medical Imaging Science, Sydney School of Health Sciences, Faculty of Medicine and Health University of Sydney Camperdown New South Wales Australia

## Abstract

Our article comments on the enduring impact of COVID‐19 in medical imaging. The emotional impact on COVID‐19 is well reported in articles published at JMRS. This editorial covers the qualitative and quantitative structured templates now used for the reporting of chest x‐rays on COVID‐19.

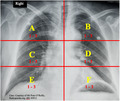

Despite the recent global easing of COVID‐19 restrictions, the SARS‐CoV‐02 virus and its numerous variants still feature prominently in public discourse. From the debates on the efficacy of vaccinations to the prognostications of future COVID variants, our world is saturated with commentaries that can leave some with a sense of vulnerability. As an example, the *New York Times* on 5^th^ May 2022 reported that a new sub‐variant spreading rapidly, named BA.2.12., is destined to become the dominant strain within the United States in 3 weeks.^1^

The disease process of the SARS‐CoV‐02 virus can manifest in a range of acute lung diseases, typically seen as pneumonia but also diagnosed at times as adult respiratory distress syndrome (ARDS) and sepsis.^2^, ^3^ Further, with 15–20% of all COVID‐19 patients demonstrating some level of lung disease, it is axiomatic that chest imaging would have quickly become a vital tool in COVID‐19 diagnosis and management, with radiographers seen in the frontline of this pandemic in their imaging roles.^4^

With that in mind, it is timely that this edition of the *Journal of Medical Radiation Sciences (JMRS)* includes an excellent article on the impact of COVID‐19 on Western Australian medical imaging clinical practices and diagnostic radiographers (hereafter termed radiographer).^5^ A section of this article examines the perceptions from the radiographer's point of view as to whether the work environment offers an adequate level of support for the psychological impact of SARS‐CoV‐02. This information is very useful and should encourage each radiographer that may be exposed to SARS‐CoV‐02 within their clinical roles to take a periodic time of reflection and share those thoughts regularly with others. Similar opinions were well expressed in the previous editorial in the *JMRS* by Smith and Dhillan.^6^ Their editorial outlined ways in which student radiographers can personally cope and build an emotional resistance to the various clinical environments exposed to the risk of SARS‐CoV‐02 by using regular communication and tailored support.^6^

From an imaging point of view, the COVID‐19 pandemic has prompted research investigating how best to report COVID‐19 chest x‐rays. With the worldwide spread of SARS‐CoV‐2 and its variants, the need for standardised radiographic chest reporting instruments has been advocated by many international radiologic societies.^7^, ^8^ Templates for chest x‐ray (CXR) and chest computed tomography (CT) reporting can provide several advantages, such as a concise and uniform descriptive language that all health professionals can understand, the ability to decrease reporting variability and ambiguity, and facilitation of improved clinical integration via the principle of a common language.^9^, ^10^

For CXR templates, there are generally two types of structured reports used as follows: The *qualitative* structured report that uses descriptive terms to explain the presence, degree of pathology or absence of radiological signs,^6^ and the *quantitative* structured report, where the radiograph is divided into anatomical sections and a numerical scale is applied to evaluate the degree of pathology seen in each section^.^ Each lung zone is then combined to give a total score, which correlates to a level of disease severity and/or anatomical involvement.^10^

An example of the *qualitative* method of structured reporting can be found in the research undertaken by Yates et al. where suspected COVID‐19 pneumonia was radiologically assessed using a structured reporting tool.^11^ Five qualitative criteria of characteristic, high suspicion, indeterminate, unlikely and normal were applied to 582 patients. The conclusion determined that chest x‐ray levels of SARS‐Cov‐02 infection correlate well with the five criteria employed and can also be useful in identifying new cases of COVID‐19 employing this qualitative method.^11^

Moreover, a study by Vespro et al. used a qualitative method to determine the presence of three lung parenchymal abnormalities.^12^ These were the appearance of ground‐glass opacity (GGO), increased opacity described commonly as consolidation and a reticular pattern which applies to the appearance of numerous small linear opacities not unlike netting. These appearances were subsequently described in the most common regions of the lungs which were laterality or bilaterality, central or peripheral and the superior or inferior location in the lungs.^12^

Alternatively, an example of the *quantitative* model of COVID‐19 chest x‐ray analysis is shown in the study by Borghesi and Maroldi, which examined a cohort of 100 patients and found the scoring system, named the BRIXIA method, was able to monitor the severity and progression of SARS‐CoV‐02.^13^ The BRIXIA system has been used by other researchers as the most reliable method to quantify levels of SARS‐CoV‐2 in those infected patients.^14^ Essentially, this method involves dividing the lungs into six zones on a postero‐anterior CXR. As is shown in Figure 1, *Line A* is drawn at the level of the inferior wall of the aortic arch. *Line B* is drawn at the level of the inferior wall of the right inferior pulmonary vein and the third vertical line divides the lungs into six compartments. Each region is given a score of 1 to 3 indicating increasing levels of infection by the higher number. The highest score possible in the BRIXIA method is 6 X 3 = 18.

**Figure 1:**
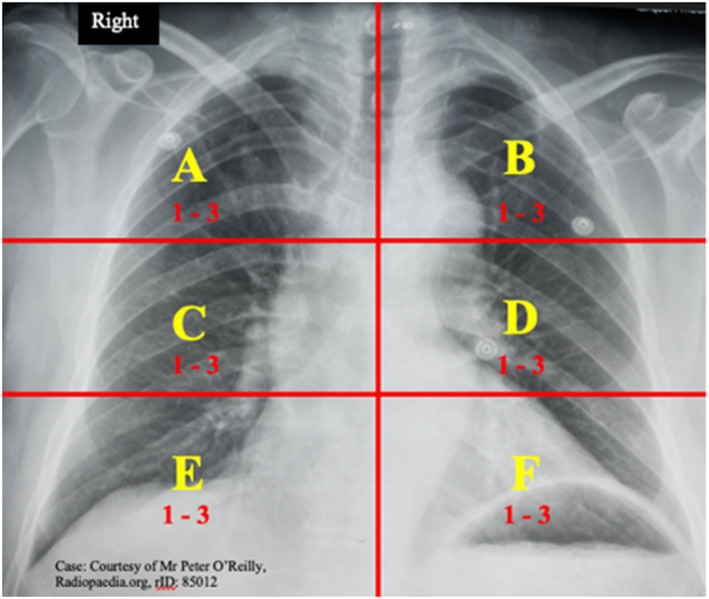
X‐ray showing the six regions used in the BRIXIA method. [Colour figure can be viewed at wileyonlinelibrary.com]

From our academic perspective, we are undertaking research in part, into the utility of these structured reporting instruments in the CXR of those patients with COVID‐19. Data assessed to this point suggest both qualitative and quantitative templates are very useful tools to not only assess the worsening of the infection at any given point in time but can also correlate with outcomes for the patient such as admission to high dependency units or intubation. This data adds extremely important information for clinicians to manage those COVID‐19 patients from admission to hospital and beyond and it is prudent that radiological research continues to examine best practice reporting methods for those patients with COVID‐19 that will aid treatment.

We would like to conclude this editorial by encouraging the many radiographers working in the public health system or private practice roles to consider broadening their interests in medical radiation science to include research. Our profession is a dynamic and contemporaneous one where we see a true nexus of the clinical and information technology in its many forms. We are well‐positioned to work with these new technologies that challenge medical imaging science to adapt to a new way of carrying out clinical practice. Take that journey in your work to embrace new ideas and be adventurous—there is much to contribute to.

## References

[1] The New York Times https://www.nytimes.com/live/2022/05/04/world/covid‐19‐mandates‐vaccine‐cases

[2] Yu N , Shen C , Yu Y , Dang M , Cai S , Guo Y . Lung involvement in patients with coronavirus disease‐19 (COVID‐19): a retrospective study based on quantitative CT findings. Chin J Acad Radiol 2020; 3: 102–7. 32395696 10.1007/s42058-020-00034-2 PMC7211979

[3] Gibson PG , Qin L , Puah SH . COVID‐19 acute respiratory distress syndrome (ARDS): clinical features and differences from typical pre‐COVID‐19 ARDS. Med J Austr 2020; 213: 54–6. 10.5694/mja2.50674 PMC7361309 32572965

[4] Naylor S , Booth S , Harvey‐Lloyd J , Strudwick R . Experiences of diagnostic radiographers through the Covid‐19 pandemic. Radiography 2022; 28: 187–92. 34736824 10.1016/j.radi.2021.10.016 PMC8552557

[5] Dann C , Sun Z . The impact of COVID‐19 on Western Australian medical imaging clinical practice and workplace. J Med Radiat Sci 2022; 69: 299–308. 10.1002/jmrs.594 35555866 PMC9348032

[6] Smith SK , Dhillon HM . Recovering and rebuilding after COVID‐19: What are the best ways to support medical radiation science students? J Med Radiat Sci 2021; 68: 339–41. 34687168 10.1002/jmrs.553 PMC8655755

[7] Rodrigues JCL , Hare SS , Edey A , et al. An update on COVID‐19 for the radiologist‐A British society of Thoracic Imaging statement. Clin Radiol 2020; 75: 323–5. 32216962 10.1016/j.crad.2020.03.003 PMC7138157

[8] Rubin GD , Ryerson CJ , Haramati LB , et al. The role of chest imaging in patient management during the COVID‐19 pandemic: a multinational consensus statement from the Fleischner Society. Radiology 2020; 296: 172–80. 32255413 10.1148/radiol.2020201365 PMC7233395

[9] Stephanie S , Shum T , Cleveland H , et al. Determinants of Chest Radiography Sensitivity for COVID‐19: A Multi‐Institutional Study in the United States. Radiol Cardiothoracic Imaging 2020; 2: e200337. 33778628 10.1148/ryct.2020200337 PMC7605075

[10] Balbi M , Caroli A , Corsi A , et al. Chest X‐ray for predicting mortality and the need for ventilatory support in COVID‐19 patients presenting to the emergency department. Eur Radiol 2021; 31: 1999–2012. 33033861 10.1007/s00330-020-07270-1 PMC7543667

[11] Yates A , Dempsey PJ , Vencken S , MacMahon PJ , Hutchinson BD . Structured reporting in portable chest radiographs: An essential tool in the diagnosis of COVID‐19. Eur J Radiol 2021; 134: 109414. 33246271 10.1016/j.ejrad.2020.109414 PMC7657021

[12] Vespro V , Andrisani MC , Fusco S , et al. Chest X‐ray findings in a large cohort of 1117 patients with SARS‐CoV‐2 infection: a multicenter study during COVID‐19 outbreak in Italy. Intern Emerg Med 2021; 16: 1173–81. 33216258 10.1007/s11739-020-02561-3 PMC7677441

[13] Borghesi A , Maroldi R . COVID‐19 outbreak in Italy: experimental chest X‐ray scoring system for quantifying and monitoring disease progression. Radiol Med 2020; 125: 509–13. 32358689 10.1007/s11547-020-01200-3 PMC7194501

[14] Gurtoo A , Agrawal A , Prakash A , et al. The Syndromic Spectrum of COVID‐19 and Correlates of Admission Parameters with Severity‐outcome Gradients: A Retrospective Study. J Assoc Physicians India 2020; 68: 43–8. 33247642

